# Supplementary material for: A simple model of co-emergence of grid and place fields
Source: ArXiv. 2026 May 20:arXiv:2605.21356v1. Preprint. [Version 1] (PMC13229076)
Supplement: Supplement 1 [file NIHPP2605.21356v1-supplement-1.pdf]

S Supplementary material

S.1 Continuous-time RNN dynamics

The discrete-time update in Eq. 1 is derived from the following continuous-time neural dynamics:

$$\tau \frac{dh_i(t)}{dt} = -h_i(t) + \sum_{j=1}^{N} W_{ij}\, \phi\big(h_j(t)\big) + \sum_{k=1}^{d_{\text{in}}} B_{ik}\, u_k(t) + b_i, \tag{S1}$$

where τ is the membrane time constant. Applying Euler discretization with step Δt and defining the leak rate $\alpha = \Delta t/\tau$, we obtain the update rule used in the main text. Throughout this work we use $\phi(\cdot) = \text{softplus}(\cdot)$ as the firing-rate nonlinearity, which provides a smooth non-negative activation. Biases b_i are fixed at zero.

S.2 Excitatory-inhibitory structure and homeostatic synaptic scaling

Sign assignment. The recurrent matrix $W \in \mathbb{R}^{N\times N}$ acts on a hidden vector of size $N = N_{\text{input-drv}} + N_{\text{rec-drv}}$, with $N_{\text{input-drv}} = N_{\text{rec-drv}} = 1024$ in all main experiments. Each column j of W (the outgoing weights of neuron j) is constrained to a single sign: 80% of neurons are randomly assigned to be excitatory (sign $+1$) and 20% inhibitory (sign -1). The sign vector $s \in \{-1, +1\}^N$ is drawn at initialization and held fixed throughout the training. We parameterize the recurrent matrix as

$$W_{ij} = |M_{ij}|\, s_j, \tag{S2}$$

where $M \in \mathbb{R}^{N\times N}$ is a learnable magnitude matrix. Because only $|M|$ enters Eq. S2, the sign of M_{ij} is irrelevant and the column sign s_j is preserved through every gradient step, enforcing Dale's law without projection.

Initialization. The magnitude matrix M is initialized as in Algorithm 1: sample a Gaussian matrix, apply Dale signs columnwise, normalize the spectral radius to 1, and store the magnitudes into M. For each neuron j, we record its total outgoing synaptic strength $c_j^* = \sum_i M_{ij}$ for the homeostatic scaling (Eq. S3). We describe the motivation for this homeostatic constraint and its role during training in the next section.

Algorithm 1 Recurrent weight initialization

1: Sample $\tilde{W}_{ij} \sim \mathcal{N}(0, 1/N)$
2: $\tilde{W}_{ij} \leftarrow s_j\, |\tilde{W}_{ij}|$ # apply Dale signs
3: $\tilde{W} \leftarrow \tilde{W}/\rho(\tilde{W})$ # normalize spectral radius to 1
4: $M_{ij} \leftarrow |\tilde{W}_{ij}|$ # store magnitudes; signs reapplied via Eq. S2
5: $c_j^* \leftarrow \sum_i M_{ij}$ # total outgoing synaptic strength of neuron j

Homeostatic synaptic scaling. Back-propagation in the RNN may update synaptic weights much faster than biological plasticity would allow, which can destabilize the training dynamics. To mitigate this, after each weight update, we apply a normalization that softly pulls each neuron's total outgoing synaptic strength toward its initial value:

$$M_{ij} \leftarrow M_{ij} \left(\frac{c_j^*}{\sum_k M_{kj}} \right)^{\eta}, \tag{S3}$$

with $\eta = 10^{-3}$. This regularization does not prevent co-emergence, but without it the network often fails to learn new environments and instead overfits to the first room.

Multiplicative firing-rate noise. Previous studies have suggested that place cells may emerge from denoising corrupted sensory input [11]. We consider two sources of such corruption. The first kind is partial occlusion of sensory cues during navigation. We model this by randomly masking sensory input channels independently across time and sensory dimensions, replacing masked values with the mean value of the corresponding sensory channel. The second is stochasticity in neural activity, this models cortical firing which is approximately Poisson-like with rate-dependent variance. Since our

model operates on firing rates rather than explicit spikes, we approximate this variability by adding Gaussian noise whose magnitude depends on the firing rate:

$$\tilde{h}_i \leftarrow \max\Big(0,\ h_i + \sqrt{\sigma_n h_i + \epsilon}\,\xi_i\Big), \quad \xi_i \sim \mathcal{N}(0,1), \tag{S4}$$

where σ_n is the noise level. Clipping enforces non-negativity of firing rates in our model, since actual spike counts are strictly non-negative.

Input and output projections. In our network, we split the hidden layer into two regions: (i) where neurons are directly driven by the sensory and motion input signal to predict the next sensory observation, and (ii) where neurons receive input only indirectly from the first region. We refer to the first region as the sensory-driven region, and the second as recurrent-driven region. The sensory-driven population receives input $u_t = [\tilde{o}_t;\, m_t] \in \mathbb{R}^{d_{\text{in}}}$ with $d_{\text{in}} = d_{\text{obs}} + d_{\text{motion}}$ through an input projection matrix $B \in \mathbb{R}^{N_{\text{input-drv}} \times d_{\text{in}}}$. The free population receives no external input. The hidden state evolves according to Eq. 1:

$$h_{i,t+1} = (1-\alpha)\, h_{i,t} + \alpha\Big(\textstyle\sum_{j=1}^{N} W_{ij}\, \phi(h_{j,t}) + \sum_{k=1}^{d_{\text{in}}} B_{ik}\, u_{k,t} + b_i\Big), \tag{S5}$$

After this deterministic recurrent update, we apply the firing-rate-dependent noise in Eq. S4 to each hidden unit. Predictions are read out from the sensory-driven population using a linear readout projection matrix: $\hat{o}_t = C\, h_t^{\text{sens-drv}}$. The network is trained to minimize the error of predicting the next observation: $\sum_t \|\hat{o}_t - o_{t+1}\|_2^2$.

S.3 Simulating sensory observations

Boundary-aware sensory input. We simulate sensory observations using a method similar to [11]. As they suggest, animals receive sensory experiences that change smoothly with location. To model this, we use random Gaussian fields for each sensory channel. Along each sensory channel, the signal varies smoothly across spatial locations, generating a smooth sensory response map of dimension $W \times H$, where W and H are the dimensions of the environment in pixels, respectively. We suppose that an animal has multiple such smooth spatial channels; together, these channels construct a sensory response map of dimension $d_{\text{sens}} \times W \times H$, where the sensory input at each location is defined by its location-specific sensory vector. The authors of [11] first initialize each sensory channel's spatial response map as a random Gaussian field, and then convolve the field with a 2D Gaussian smoothing kernel where the kernel width is σ. However, this method does not respect environmental boundaries. Near a boundary, if σ is larger than the spatial width of a wall, then the 2D Gaussian smoothing kernel can blend sensory observations across the wall.

To resolve the above issue, we replace Gaussian smoothing with a boundary-aware diffusion process that runs for n_{iter} steps. At each step, we apply a 3×3 box filter at each location that averages the sensory responses at neighboring locations that lie in free space. This prevents smoothing across walls and respects environmental boundaries. We set $n_{\text{iter}} = \lceil 1.5\sigma^2 \rceil$ because since one step of a 3×3 box filter has variance $2/3$ along each spatial dimension; so after n steps the effective Gaussian width is $\sqrt{2n/3}$. This gives $n \approx 1.5\sigma^2$ for a smoothing width σ.

Populations. We use four sensory input populations with different smoothing widths $\sigma \in \{6, 8, 10, 12\}$ cm, each containing $N_{\text{cell}} = 256$ cells, giving a total sensory dimension of $d_{\text{obs}} = 1024$. Using multiple smoothing widths allows the sensory observations to contain both fine- and coarse-scale spatial features.

S.4 Trajectory generation

Trajectory generation. We simulate animal trajectories as a smooth random walk. At each timestep t, the agent maintains a current speed v_t and unit movement direction $\mathbf{d}_t$, together with a target speed v_t^* and target direction $\mathbf{d}_t^*$ unit vector. The target variables represent the speed and direction that the agent is currently trying to move toward. Instead of choosing a completely new velocity at every timestep, the agent only occasionally resamples these targets: v_t^* is redrawn with probability p_v and $\mathbf{d}_t^*$ is redrawn with probability p_d. This way, the agent tends to continue moving in a similar way for several timesteps before changing its intended motion. Speed targets are sampled from a log-normal distribution with mean μ_{spd} and standard deviation σ_{spd} (speed is positive). Directions are sampled as random unit vectors in $\mathbb{R}^2$ or $\mathbb{R}^3$ depending on the simulation environment. To

avoid abrupt changes when a new target is sampled, the current speed and direction are gradually updated toward their targets using exponential moving averages: $v_{t+1} = (1 - \alpha_v)v_t + \alpha_v v_t^*$, and $\mathbf{d}_{t+1} = \text{normalize}((1 - \alpha_d)\mathbf{d}_t + \alpha_d \mathbf{d}_t^*)$. Here, α_v and α_d control how quickly the simulated agent adapts to the newly sampled target speed and direction.

Boundary avoidance. To prevent the agent from running into walls or getting stuck near boundaries, we apply a soft boundary avoidance near walls. Before trajectory generation, we compute a distance-to-wall map $d(x)$, which gives the distance from each location x to the nearest wall and a local wall-normal direction $\mathbf{n}(x)$ which points away from the wall. We also define an avoidance strength $c(x)$ which is large near walls and decays as the agent moves farther away from them. When the agent is far from any wall, its movement direction $\mathbf{d}_t$ is unchanged. When the agent is close to a wall and moving towards it, we compute the wall-parallel direction by removing the component of $\mathbf{d}_t$ that points into the wall. We then blend the current direction with this wall-parallel direction, with a blending weight β_t that increases when the agent is closer to the wall and when its speed is larger. This softly turns the agent along the boundary instead of abruptly reflecting it, producing smooth wall-following trajectories in both 2D and 3D environments.

Training along long trajectories. Previous RNN studies [8, 11, 13] train their models with backpropagation through time (BPTT) on short trajectory segments, typically around 20–40 simulation steps or approximately 1–2 s. In their setup, the network is updated using inputs sampled from short trajectory fragments; the simulated agent is relocated to a new position after each update. The authors in [8] initialize the network hidden state with a small multilayer perceptron after each location reset, while the authors in [11] initialize the hidden state to zero. Our model is also trained with BPTT. But we realized that such reset-based training schemes do not allow studying place-cell and grid-cell behavior over longer continuous trajectories. This is especially true in experiments such as the hairpin maze. We resolve this issue by detaching the last hidden state from the previous training segment and use it to initialize the first hidden state of the current training segment. This is the appropriate way to implement truncated BPTT and it allows the model to be trained on short segments while preserving continuous trajectory dynamics across updates.

S.5 Sensory masking

As discussed in Section S.1, we simulate noisy sensory inputs by masking parts of it by the mean sensory response. Masking is applied independently across the batch, timesteps and sensory dimensions. At each timestep, each sensory dimension is independently masked with probability r_{mask}, where r_{mask} denotes the masking ratio. Masked entries are replaced by the corresponding cell's mean firing rate, computed over the arena, while unmasked entries keep their original sensory value. Using the per-cell mean rather than zero prevents the network from treating masked entries as an artificial zero-valued cue.

S.6 Collecting ratemaps during training

Experimentally measured ratemaps are obtained while the animal explores actively. Thus, instead of freezing the model and evaluating it in a separate testing phase, we continuously compute the firing rate of different cells using the trajectories sampled during training. At each optimization step, we collect hidden states from all batches and timesteps, together with the corresponding agent coordinates. The coordinates are rounded to the nearest spatial bin. For each visited bin, we compute each hidden unit's mean firing rate over all visits to that bin. This gives a ratemap estimate over the subset of spatial bins visited during the current optimization step. To aggregate ratemaps over training, we maintain one running ratemap for each hidden unit. At each update step, only the spatial bins visited by the current trajectories are updated. For those bins, the ratemap values from previous updates are combined with the average firing rates computed at the current update step using an exponential moving average with decay $\gamma = 0.995$. Spatial bins that are not visited keep their previous values and bins that have never been visited are marked as undefined. This allows ratemaps to be accumulated smoothly throughout training while giving slightly higher weight to more recent activity. With $\gamma = 0.995$, the ratemap retains an effective memory of approximately 200 update steps for regularly visited bins.

Table S1: Default training and architectural parameters.

**Parameter**	**Value**	**Description**
$N_{\text{input-drv}}$	1024	Sensory-driven population size
$N_{\text{rec-drv}}$	1024	Free population size
$\phi(\cdot)$	softplus	Firing-rate nonlinearity
EI ratio	0.8 / 0.2	Excitatory / inhibitory fraction
α	0.5	Default leak rate (sigmoid-gated)
σ_n	0.8	Default firing-rate noise level
b_i	0 (fixed)	Bias term in Eq. 1
η	10^{-3}	Homeostatic scaling exponent
optimizer	AdamW	
learning_rate	$5 \cdot 10^{-4}$	
batch_size	256	Each batch is one batch of trajectories
traj_duration	10	Backpropagation window
gradient_clip	1.0	L2 norm cap on parameter gradients
Δt	1/20 s	Simulation timestep
$\mu_{\text{spd}}, \sigma_{\text{spd}}$	40, 20 cm/s	Log-normal speed mean and std
α_v, α_d	0.8, 0.2	EMA smoothing for speed and direction
p_v, p_d	0.2, 0.1	Per-step target switch probabilities
σ_{cell}	$\{6, 8, 10, 12\}$	Sensory cells smoothing widths
N_{cell}	256 per population	Cells per sensory population
r_{mask}	0.2	Default sensory mask ratio

S.7 Default training parameters

Unless otherwise stated, all reported runs use the parameters in Table S1. Per-experiment overrides are listed in their respective sections.

S.8 Cell classification

We classify cells into three types: *place*, *grid* or *other spatial*, based on per-cell scores computed from each ratemap.

Classifying activated spatial cells. Since both grid cells and place cells are strongly modulated by an animal's spatial locations, we first filter out cells that are active and have spatial information content above 0.2. We define a cell as active if it has a mean firing rate $\bar{r}_i \leq 0.1$. The spatial information content is

$$\text{SIC}_i = \sum_{\mathbf{x}} p(\mathbf{x}) \frac{R_i(\mathbf{x})}{\bar{r}_i} \log_2 \frac{R_i(\mathbf{x})}{\bar{r}_i}, \quad \text{(S6)}$$

where $p(\mathbf{x})$ is the empirical occupancy of spatial bin $\mathbf{x}$. Cells with $\text{SIC}_i > 0.2$ are treated as spatial cells.

Place-cell classification. We next test whether selected spatial cells are place cells. Previous studies often use SIC alone to identify place cells. However, because our model contains place cells, grid cells, and other spatially tuned cells within the same hidden layer, SIC alone may not be sufficient for robust classification. We therefore add two firing-field locality criteria for place-cell classification. Specifically, a place cell should contain only a small number of spatially localized firing fields, rather than many repeated peaks. In addition, its firing should be concentrated to a small spatial region. To measure this, we first threshold each ratemap at 0.3 of its maximum firing rate. After thresholding, each connected group of spatial bins above the threshold is identified as a firing field. We classify a cell as a place cell if it has fewer than 3 such firing fields and if its largest field contains at least 35% of the cell's total firing mass.

Spatial autocorrelogram. To classify grid cells, previous studies have used autocorrelograms to reveal the characteristic spatial periodicity of grid-cell firing [1]. In this study, we use the same spatial autocorrelogram computation method as that of [1]. Specifically, for each ratemap $R_i(\mathbf{x})$, we compute a 2D spatial autocorrelogram to measure whether the firing fields form a regular periodic structure. For each spatial lag $\boldsymbol{\tau} = (\tau_x, \tau_y)$, we compare the ratemap with a shifted copy of itself, $R_i(\mathbf{x} - \boldsymbol{\tau})$. The correlation is computed only over spatial bins where both $R_i(\mathbf{x})$ and $R_i(\mathbf{x} - \boldsymbol{\tau})$ are valid, which corrects for edge effects and unvisited locations. Following this standard sample-correlation formulation, the autocorrelation at lag $\boldsymbol{\tau} = (\tau_x, \tau_y)$ for cell i, with ratemap $R \equiv R_i$, is

$$A(\boldsymbol{\tau}) = \frac{n \sum R(\mathbf{x}) R(\mathbf{x} - \boldsymbol{\tau}) - \sum R(\mathbf{x}) \sum R(\mathbf{x} - \boldsymbol{\tau})}{\sqrt{n \sum R(\mathbf{x})^2 - \left(\sum R(\mathbf{x})\right)^2} \sqrt{n \sum R(\mathbf{x} - \boldsymbol{\tau})^2 - \left(\sum R(\mathbf{x} - \boldsymbol{\tau})\right)^2}}, \tag{S7}$$

where all sums are taken over the n spatial bins for which both $R(\mathbf{x})$ and $R(\mathbf{x} - \boldsymbol{\tau})$ are valid. Lags with too few overlapping valid bins are not evaluated. The resulting autocorrelogram is shifted so that the zero-lag term is centered, producing a $(2H - 1) \times (2W - 1)$ map for an $H \times W$ ratemap.

Grid score. The "grid score" measures whether a cell has the six-fold rotational symmetry characteristic of grid cells. Following the standard grid-score procedure [45], we take a centered crop of the autocorrelogram computed above with radius $r = 60$ bins. We exclude the small central peak around zero lag, since this peak appears for all cells, and also exclude a narrow edge band to avoid boundary artifacts. We then compute the Pearson correlation between the autocorrelogram and a rotated copy of itself. Let ρ_θ denote this correlation after rotation by θ degrees. The grid score is defined as

$$\mathrm{GS}_i = \min(\rho_{60}, \rho_{120}) - \max(\rho_{30}, \rho_{90}, \rho_{150}). \tag{S8}$$

A hexagonal grid should have high correlation after $60°$ and $120°$ rotations, but lower correlation after $30°$, $90°$, and $150°$ rotations.

Note that we classify grid cells after place-cell classification. This ordering is unlikely to remove valid grid cells, because our place-cell criterion requires fewer than 3 firing fields, whereas a valid grid cell should contain multiple periodically repeated fields. Among the remaining spatially tuned cells, we classify a cell as a grid cell if it passes the grid-score threshold. We use $\mathrm{GS}_i > 0.3$ for all analyses, except for the parameter-sweep result in Figure 2F. For the parameter sweep, we use a more lenient threshold of $\mathrm{GS}_i > 0.1$, so that the summary better reflects how grid-like periodic patterns change across the swept parameters. We also require $\min(\rho_{60}, \rho_{120}) > 0$, ensuring that a positive grid score is supported by positive correlations at the grid-symmetric rotations. Finally, we require at least 4 firing fields, which prevents single-peaked or weakly localized spatial cells from being classified as grid cells.

Other spatial cells. Spatial cells that do not satisfy either the place or grid criteria are labelled *other spatial*. These are cells with structured but non-localized and non-hexagonal spatial tuning (e.g., stripe-like, multi-peaked irregular fields, or border-like).

S.9 Parameter sweep details

Sweep grid. The parameter sweep in the main text (Figure 2F) covers a $10 \times 10 \times 10$ grid over the leak rate, firing-rate noise level and sensory mask ratio:

$$\alpha \in \{0.1, 0.2, \ldots, 1.0\}, \quad \sigma_n \in \{0, 0.05, \ldots, 0.45\}, \quad r_{\text{mask}} \in \{0, 0.1, \ldots, 0.9\}. \tag{S9}$$

For each configuration, we train the network for 200,000 weight updates in the $220\,\text{cm} \times 220\,\text{cm}$ square arena while holding all other parameters at their default values (Table S1). We use a different random seed for each training condition to test robustness across stochastic components of the setup, including the sensory response fields, trajectories, and network initialization. We use only one random seed for each configuration (different for each configuration). When summarizing results along each marginal axis, each value aggregates over 100 sweep conditions, providing implicit averaging over the other parameters and random seeds.

S.10 Two-room wall removal experiment

In this experiment, two $180 \times 150\,\text{cm}^2$ rectangular rooms are placed side-by-side and separated by a thin internal wall of thickness equal to twice the simulator's 5-cm border, giving a merged arena of size $180 \times 310\,\text{cm}^2$. We train the network over three phases, each with 100,000 network updates:

1. Room 0 (left sub-room). The agent is spawned and confined to the left compartment.
2. Room 1 (right sub-room). The agent is respawned in the right compartment.
3. Merged arena. The internal wall is removed, and the agent is allowed to move freely through the full arena.

At initialization, we first sample a random Gaussian noise field. We then generate the sensory response maps for the two rooms using the boundary-aware diffusion smoothing method described in Section S.3. Because this diffusion process respects environmental boundaries, sensory values are not smoothed across the wall separating the two rooms. As a result, the sensory response maps on the two sides of the internal wall can differ sharply. During the first two phases, the agent is spawned in the corresponding room, and its trajectory is constrained to be within that room because the two rooms are not connected.

After the agent has explored both rooms, we remove the internal wall. Starting from the same initial Gaussian noise field, we re-apply the diffusion-based smoothing process on the merged arena. After this re-smoothing, sensory responses near the original wall location become smoothly connected across the two formerly separated rooms. Since the diffusion process only acts locally, sensory responses at locations far from the removed wall are expected to remain similar to their values before wall removal.

Across all three phases, we use the parameters in Table S1, with $\alpha = 0.5$, $\sigma_n = 0.8$, and $r_{\text{mask}} = 0$. We maintain a ratemap aggregator over the full merged arena. During phases 0 and 1, only the bins visited in the active room are updated, while bins in the other room remain unchanged. At the beginning of phase 2, we reset the ratemap aggregation so that the merged-arena ratemaps reflect only the post-wall-removal experience.

S.11 Hairpin maze experiment

The hairpin arena contains 10 parallel corridors connected by alternating turn gaps, forming a continuous serpentine path. Its bounding box is matched to the open arena, so the same raw sensory field can be evaluated in both environments after applying boundary-aware diffusion smoothing. To construct the sensory inputs, we first generate one raw Gaussian noise field for each sensory population. We then apply the boundary-aware diffusion process separately in the open arena and in the hairpin maze. Because the raw field is shared, corresponding locations have similar sensory values away from the inserted walls. In the hairpin maze, however, diffusion is blocked by corridor walls, so sensory similarity follows the corridor structure rather than passing through walls.

In the open-arena phases, trajectories are generated by the default smooth random-walk generator. In the hairpin phases, we use a scripted serpentine controller that drives the agent through the corridors one lap at a time: left-to-right in the second phase (Figure 3B, MazeLR) and right-to-left in the third phase (Figure 3B, MazeRL). The hidden state is reset to zero at each lap boundary, so successive laps form independent trials. At every step, speed is sampled from the same log-normal distribution used by the open-arena controller. We also add small random lateral offsets within each corridor, together with a perpendicular drift, so that the agent samples the full corridor width rather than only following the centerline.

Following [31], we train the model across four phases: open arena, hairpin forward, hairpin backward, and open arena again. During each phase, we update the network for $100,000$ steps. We reset the accumulated ratemap whenever we shift the training phase, so each phase's ratemaps reflect only cell activity collected during that phase.

S.12 Connected-rooms experiment

We use two $120 \times 120\,\text{cm}^2$ compartments separated by an $8\,\text{cm}$ dividing wall, with a $248 \times 70\,\text{cm}^2$ return corridor along the north side. Each compartment connects to the corridor through a single $80\,\text{cm}$ opening centered on its north wall, so the corridor and the two compartments form one continuous walkable region. The fully connected arena is treated as a single environment during both sensory-field generation and training. Therefore, the sensory field changes smoothly across the corridor. We train the network with $800,000$ update steps, allowing grid-cell-like representations to stabilize while also tracking their drift over training.

S.13 Three-dimensional traversal experiments

For the 3D experiments, we use a cubic arena of size $97 \times 97 \times 97$ bins, with a 5-voxel border giving a 107^3 ambient map. The four sensory populations use 3D smoothing widths $\sigma \in \{8, 10, 12, 14\}$, each containing 256 cells. The boundary-aware diffusion process described in Section S.3 is applied with a volumetric $3 \times 3 \times 3$ kernel. The motion input is 4-dimensional, consisting of a 3D unit movement direction and a scalar speed.

In the random-fly traversal experiment, the agent moves freely through the volume using the same smooth trajectory generator and boundary-avoidance scheme as in 2D. We use $\mu_{\text{spd}} = 30$, $\sigma_{\text{spd}} = 10$, and $\alpha_v = 0.5$, and train the model for 800,000 steps with firing-rate noise $\sigma_n = 0.8$. Under this traversal, place-like cells develop localized 3D firing fields, while grid-like cells develop locally ordered volumetric firing fields, consistent with the partially ordered 3D grid patterns reported in flying bats.

S.14 Robustness of co-emergence

S.14.1 Region ratio

The default network uses $N_{\text{sens-drv}} = N_{\text{rec-drv}} = 1024$. To examine whether this exact split affects co-emergence, we trained four additional models with different sensory-driven/recurrent-driven ratios: $(N_{\text{sens-drv}}, N_{\text{rec-drv}}) \in \{(512, 1536), (768, 1280), (1280, 768), (1536, 512)\}$, while holding the total number of hidden units fixed at $N = 2048$.

Across all four models, both place cells and grid cells emerged consistently. The corresponding numbers of classified place/grid cells were $263/68$, $302/43$, $307/94$, and $313/12$, respectively. Although our main experiments were conducted in the $1024/1024$ setting for purposes of clarity, this experiment shows that co-emergence is not specific to that case. The reduced grid-cell count in the $1536/512$ split suggests that having too few recurrent-driven units can limit grid-cell emergence, but the qualitative presence of both cell types remains consistent across the tested partitions.

S.14.2 Disabling homeostatic synaptic scaling

Setting $\eta = 0$ in Eq. S3 removes the column-wise outgoing-budget rescaling. Over short training horizons ($\leq 50{,}000$ steps), grid- and place-like responses still emerge qualitatively. However, over longer training horizons, the recurrent dynamics gradually drift: units that initially exhibit hexagonally arranged firing fields lose contrast over time, with firing-field centers becoming progressively weaker until the periodic structure is no longer visually apparent. We also found that, without homeostatic scaling, switching to a new room can produce infinite losses, possibly because the network has overfit to the preceding room. Homeostatic scaling therefore acts as a soft stabilizer that preserves the co-emergent operating regime over extended training, rather than being strictly required for the initial formation of grid- or place-like responses.
